# Remarkable response to crizotinib in a patient with advanced lung adenocarcinoma harboring the MPRIP-ROS1 fusion gene: A case report

**DOI:** 10.1016/j.rmcr.2025.102243

**Published:** 2025-06-25

**Authors:** Yasuyuki Kishikawa, Kohei Otsubo, Daisuke Shibahara, Yoshimasa Shiraishi, Yasuto Yoneshima, Eiji Iwama, Isamu Okamoto

**Affiliations:** Department of Respiratory Medicine, Graduate School of Medical Sciences, Kyushu University, 3-1-1 Maidashi, Higashi-ku, Fukuoka, 812-8582, Japan

**Keywords:** Non-small cell lung cancer, *MPRIP-ROS1* fusion, Crizotinib, Comprehensive genomic profiling

## Abstract

For patients with advanced non-small cell lung cancer (NSCLC), genetic testing is crucial to identify alterations in targetable driver genes. ROS1-tyrosine kinase inhibitors have shown efficacy against NSCLC with common *ROS1* fusion genes, but the impact of rare fusion partners on therapeutic outcomes is not well understood. Here, we describe a 75-year-old female with advanced lung adenocarcinoma who was treated with crizotinib after the identification of the extremely rare *MPRIP-ROS1* fusion. Despite stepwise dose reductions due to adverse effects, the patient exhibited a significant tumor response to crizotinib. The sustained response, even at reduced doses, highlights the potential for targeted therapies in managing NSCLC with *MPRIP-ROS1* fusion. This case also underscores the importance of comprehensive genomic profiling using hybrid capture-based next-generation sequencing to identify rare driver gene alterations that may not be detected by conventional target sequencing-based methods.

## Highlights

• Herein, we present a female patient with advanced non-small cell lung cancer harboring the *MPRIP-ROS1* fusion gene, an extremely rare ROS1 rearrangement.
• Crizotinib was remarkably effective against advanced non-small cell lung cancer associated with the *MPRIP-ROS1* fusion gene even after its dose was reduced due to adverse events.
• Hybrid capture-based comprehensive genomic profiling enables the detection of rare or previously unidentified driver gene alterations that cannot be identified via targeted sequence-based genetic testing, thereby guiding targeted therapies and improving patient outcomes.

## 1 Introduction

Lung cancer is one of the most common types of cancer and the leading cause of cancer-related mortality worldwide [1]. The recent development of molecular-targeted agents has significantly improved progression-free survival and overall survival in patients with non-small-cell lung cancer (NSCLC). Genetic testing, which can detect alterations in targetable driver genes, is recommended for patients diagnosed with advanced NSCLC [2]. Approximately 0.9 %–2.6 % of patients with NSCLC present with ROS proto-oncogene 1 receptor tyrosine kinase (*ROS1*) fusions. Further, these fusions are frequently observed in young patients, women, and never smokers. Multiple fusion partners of *ROS1* have been reported, with the most frequent being *CD74* (38 %–54 %), *EZR* (13 %–24 %), *SDC4* (9 %–13 %), and *SLC34A2* (5 %–10 %) [3]. ROS1-tyrosine kinase inhibitors (TKIs) such as crizotinib, entrectinib, and repotrectinib are effective against NSCLC harboring these common *ROS1* fusion genes and are recommended as the first-line treatment [[4], [5], [6]]. However, the impact of rare fusion partners on the efficacy of these ROS1-TKIs is not well understood. Herein, we report a case of advanced lung adenocarcinoma associated with the *MPRIP-ROS1* fusion gene, a rare *ROS1* rearrangement, detected using a comprehensive genomic profiling test in a patient with remarkable tumor response to crizotinib.

## 2 Case presentation

A 75-year-old female patient without a history of smoking presented to the hospital due to persistent cough. Chest computed tomography (CT) scan showed a mass in the middle lobe of the right lung and pleural effusion. Lung adenocarcinoma was confirmed via pleural effusion cytology, and the clinical stage was determined to be stage IVA (cT2aN3M1a). Due to the unavailability of tumor tissues, polymerase chain reaction (PCR)-based epidermal growth factor receptor (EGFR) mutation analysis was performed using pleural fluid samples. However, mutations were not detected. First-line therapy with carboplatin (CBDCA) and pemetrexed (PEM) combined with atezolizumab was initiated. After one cycle of treatment, atezolizumab was discontinued because the patient developed skin rash. However, treatment with CBDCA and PEM was continued. After four cycles of treatment, the tumor size significantly decreased, and the pleural effusion resolved. However, due to the persistence of skin rash, maintenance therapy with PEM was not administered. Twelve months after the last chemotherapeutic dose, CT scan showed progression of the primary tumor and increased pleural dissemination. Hence, the patient was referred to our hospital for second-line treatment.

After collecting tumor tissues via CT scan-guided lung biopsy, PCR-based multiplex driver gene testing was performed using the AmoyDx Pan Lung Cancer Multi-Gene PCR Panel. Alterations in the *EGFR*, *ALK*, *ROS1*, *BRAF*, *KRAS*, *RET*, *MET*, *HER2*, *and NTRK* genes were not detected. Based on a high PD-L1 tumor proportion score at 75 % based on the IHC 22C3 pharmDx assay, pembrolizumab at a dose of 200 mg was administered every 3 weeks as the second-line therapy. However, it was not effective, and the patient was diagnosed with progressive disease 3 months after the initiation of pembrolizumab treatment.

**Fig. 1:**
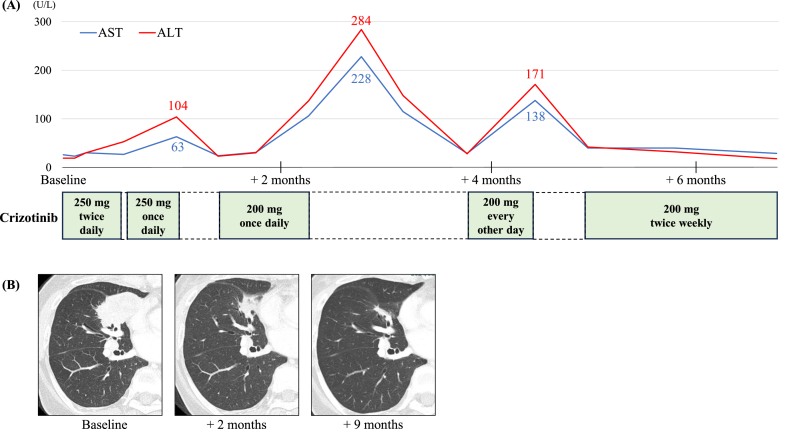
(A) Timeline of the patient's clinical treatment course. The upper graph shows the trend in aspartate aminotransferase (AST) and alanine aminotransferase (ALT) values. The lower boxes indicate the doses of crizotinib administered. The dashed blank boxes represent the periods of crizotinib withdrawal. (B) Computed tomography (CT) scan images of the primary lesions in the right middle lobe at baseline and after 2 and 9 months of crizotinib treatment.

Comprehensive genomic profiling using FoundationOne CDx revealed the presence of the *MPRIP-ROS1* fusion, a rare *ROS1* gene rearrangement. Therefore, third-line treatment with crizotinib at a dose of 250 mg twice daily was initiated. The crizotinib dose was reduced to 200 mg once daily due to nausea and elevated aspartate aminotransferase and alanine aminotransferase levels. However, CT scan showed a remarkable decrease in tumor size 2 months after the treatment initiation. The crizotinib dose was reduced stepwise as the patient had elevated aspartate aminotransferase and alanine aminotransferase levels. Nevertheless, these adverse events were eventually managed at a dose of 200 mg twice weekly (Fig. 1A). CT scan revealed sustained response 9 months after the treatment initiation, and the patient is still followed-up for crizotinib treatment (Fig. 1B).

## 3 Discussion

The ROS1 gene, located on chromosome 6q21, is composed of 44 exons, and it encodes a receptor tyrosine kinase involved in cellular proliferation and differentiation [7]. *ROS1* rearrangements occur at breakpoints within exons 32–36 or introns 31–33, retaining the ROS1 kinase domain and forming several partner genes. These rearrangements promote constitutive kinase activation, particularly when the fusion partners possess coiled-coil domains that enable the dimerization of fusion proteins. The constitutive activation of ROS1 fusion proteins drives oncogenesis in NSCLC via multiple signaling cascades, primarily the PI3K/AKT, MAPK/ERK, and STAT3 pathways [8,9].

Herein, we described *MPRIP-ROS1* fusion, which is rare, in advanced lung adenocarcinoma. To date, only one case of NSCLC harboring the MPRIP-ROS1 fusion has been reported worldwide. In this case, crizotinib that was administered for over 16 months was found to be effective [10]. In the current case, the patient had a remarkable response to crizotinib. Notably, the response was sustained even after repeated dose reductions caused by adverse effects. This finding underscores the high sensitivity of *MPRIP-ROS1* fusion-positive NSCLC to crizotinib, even at low doses, particularly in cases where dose reduction is required due to toxicity.

*MPRIP* is a regulator of RNAPII transcription [11] and a modulator of vasodilation [12] and is involved in leukocyte trafficking [13]. Fusion of *MPRIP* with the proto-oncogenes *NTRK1* [14] and *ALK* [15] in NSCLC is only reported in one case each, and both patients were responsive to crizotinib. In our case, which involved *MPRIP-ROS1* fusion, both the coiled-coil domain of *MPRIP* and the kinase domain of *ROS1* were preserved, which likely led to the dimerization and kinase activation of *ROS1* and sensitivity to crizotinib.

The approved targeted sequence-based multiplex genetic tests can detect most targetable driver gene alterations in NSCLC. However, they cannot identify extremely rare fusion genes that have not been prespecified for detection. Therefore, *MPRIP-ROS1* fusion was not identified in our case, even though major *ROS1* fusions were covered by PCR-based multiplex driver gene testing. In contrast, comprehensive hybrid capture (HC)-based next-generation sequencing (NGS) can be used to examine the whole coding sequence of oncogenes and tumor suppressor genes, thereby providing extensive genetic information on a wide array of alterations. This includes data on exon and intron mutations, gene rearrangements, and amplifications, thereby enabling the detection of rare or previously unidentified fusion genes that are not detectable on targeted sequence-based testing [16]. HC-based NGS can identify clinically actionable genomic alterations in 50 % (50/101) of patients with negative or inconclusive EGFR/ALK testing. Molecular-targeted therapy based on the result of this HC-based NGS test resulted in a favorable overall response rate at 65 %, which indicated survival benefit [17]. These results underscore the clinical utility of HC-based NGS in guiding targeted therapy selection and improving patient outcomes.

This case report shows that crizotinib is effective against *MPRIP-ROS1* fusion-positive adenocarcinoma, even with stepwise dose reductions for toxicity management. Further, it emphasizes the importance of performing HC-based comprehensive genomic profiling tests to explore the presence of rare genetic alterations in patients with NSCLC who tested negative for driver gene alterations based on conventional TC-based genetic tests, particularly in cases of adenocarcinoma not associated with a smoking history.

## CRediT authorship contribution statement

**Yasuyuki Kishikawa:** Formal analysis, Methodology, Investigation, Writing – original draft, Data curation, Writing – review & editing. **Kohei Otsubo:** Methodology, Formal analysis, Writing – original draft, Writing – review & editing, Conceptualization, Investigation, Data curation, Project administration. **Daisuke Shibahara:** Conceptualization, Writing – review & editing. **Yoshimasa Shiraishi:** Supervision, Writing – review & editing. **Yasuto Yoneshima:** Supervision, Writing – review & editing. **Eiji Iwama:** Writing – review & editing, Supervision. **Isamu Okamoto:** Writing – review & editing, Project administration, Supervision.

## Disclosure

The authors declare no conflicts of interest to declare.

## Funding sources

This research did not receive any specific grant from funding agencies in the public, commercial, or not-for-profit sectors.

## Declaration of competing interest

The authors declare that they have no known competing financial interests or personal relationships that could have appeared to influence the work reported in this paper.
